# Enhanced amygdala–anterior cingulate white matter structural connectivity in Sahaja Yoga Meditators

**DOI:** 10.1371/journal.pone.0301283

**Published:** 2024-03-28

**Authors:** Oscar Perez-Diaz, Daylín Góngora, José L. González-Mora, Katya Rubia, Alfonso Barrós-Loscertales, Sergio Elías Hernández

**Affiliations:** 1 Instituto Universitario de Neurociencia, Universidad de La Laguna, San Cristóbal de La Laguna, Tenerife, Spain; 2 Department of Microeconomics and Public Economics, Maastricht University School of Business and Economics & Maastricht University ‐ Center of Neuroeconomics, Faculty of Psychology and Neuroscience, Maastricht University, Maastricht, The Netherlands; 3 Institute of Psychiatry, Psychology and Neuroscience, King’s College London, London, United Kingdom; 4 Departamento de Psicología Básica, Clínica y Psicobiología, Universitat Jaume I, Castellón de la Plana, Castellón, Spain; Gachon University, REPUBLIC OF KOREA

## Abstract

**Objective:**

To study the white matter connections between anterior cingulate cortex, anterior insula and amygdala as key regions of the frontal-limbic network that have been related to meditation.

**Design:**

Twenty experienced practitioners of Sahaja Yoga Meditation and twenty nonmeditators matched on age, gender and education level, were scanned using Diffusion Weighted Imaging, using a 3T scanner, and their white matter connectivity was compared using diffusion tensor imaging analyses.

**Results:**

There were five white matter fiber paths in which meditators showed a larger number of tracts, two of them connecting the same area in both hemispheres: the left and right amygdalae and the left and right anterior insula; and the other three connecting left anterior cingulate with the right anterior insula, the right amygdala and the left amygdala. On the other hand, non-meditators showed larger number of tracts in two paths connecting the left anterior insula with the left amygdala, and the left anterior insula with the left anterior cingulate.

**Conclusions:**

The study shows that long-term practice of Sahaja Yoga Meditation is associated with larger white matter tracts strengthening interhemispheric connections between limbic regions and connections between cingulo-amygdalar and cingulo-insular brain regions related to top-down attentional and emotional processes as well as between top-down control functions that could potentially be related to the witness state perceived through the state of mental silence promoted with this meditation. On the other hand, reduced connectivity strength in left anterior insula in the meditation group could be associated to reduced emotional processing affecting top-down processes.

## Introduction

Meditation is becoming widely popular as an alternative way to improve life quality [1–7]. There are several ways to approach the practice of meditation, each addressing distinct objectives or needs. These may include enhancing consciousness, cultivating compassion and inner peace, pursuing transcendence/ spirituality, reducing stress, or achieving self-awareness [8–13].

To achieve these goals, meditation comprehends a huge variety of practices, most of which are based on improving attention and emotion control [13–15]. For example, focused attention meditation (FAM), involves inward reflection of observation, directing the attention on different parts of the body, or focusing on physiological processes like breathing [15]. Other techniques, such as open monitoring meditation (OMM), focus on the nonjudgmental observation of the mind and thoughts, or the surrendering of thoughts and emotions to enhance present moment awareness [15]. Most meditation techniques incorporate aspects of these methods as well as others [3, 15–18].

Sahaja Yoga Meditation (SYM) is a practice where practitioners subjectively perceive a subtle inner body composed of the energy of meditation (Kundalini), modulating centers called chakras and three energy channels, based in ancient yoga texts (a diagram with the location of the chakras, the kundalini, and their qualities according to SYM is provided as supporting information. See S1 File) [19, 20]. SYM allows meditators to perceive the state of their different chakras and attain thoughtless awareness or mental silence (MS) [19, 21, 22]. MS is a profound meditative state in which individuals have very few or no thoughts and are completely aware of their surroundings and inner self. MS is a state of high efficiency, combining calmness with alertness, where one is fully present in the here and now [21]. The phenomena related to the perception of the subtle body and MS have enhanced our scientific interest on this meditation [22–26].

While the subjective perception of the subtle body is unique of SYM, there are connections with other forms of meditation previously mentioned. For example, the interiorized attention on the different chakras could be perceived as a type of FAM. The state of MS, where meditators fully perceive each present moment with few or no thoughts, could be related with a type of OMM. Furthermore, the specific attention in the heart chakra, related with pure love and compassion, may be related with compassion meditation [27]. However, this parallelism between practices has not been studied and we have described it based on previous research [15, 28].

Research has linked different meditation types with effects on neuronal activity, larger gray matter volume (GMV) and white matter (WM) in anterior cingulate cortex (ACC) [26, 29–32]. The ACC lies between the neocortex and the limbic system and plays a pivotal role in cognitive/attention and emotion control, specifically the latter through the top-down regulation of the limbic system [27, 33]. Previously, we have associated rostral ACC, extending to medial prefrontal cortex (PFC), with SYM based on evidence for larger GMV in this region in long-term SYM practitioners [26]. Furthermore, this GMV effect was correlated with the depth of meditation or depth of MS perceived during magnetic resonance imaging (MRI) scans. Additionally, positive functional connectivity (FC) between ACC and bilateral anterior insula (AI), extending to putamen, correlated with the perceived depth of meditation inside the scanner [26].

Research on different meditation practices, such as Raja Yoga meditators [34], Integrative Body–Mind Training [29], different types of Buddhist meditations [35] or Quadrato Motor Training meditation [36], have reported WM differences in the corpus callosum (CC) of meditators. These findings align with reported GMV differences in prefrontal areas across multiple studies of meditation (for a review see [37–39]). Furthermore, recent studies have presented evidence on meditation having effects in some major WM tracts of the human brain. Many have focused on the WM tracts connecting widely reported key meditation areas, such as: Insula [40–42], Amygdala [41, 42] or ACC [29, 30, 41, 43].

Relatively little is known on the brain basis of SYM or the state of MS on emotion processing and neural circuits of cognitive control. In addition to the previously reported effects on ACC, we have found that SYM induces activation of the right inferior frontal gyrus (IFG), extending to right AI [23]. Furthermore, structural analyses showed that long-term SYM practitioners present larger GMV in the right insula-IFG, as well as in the left insula and left IFG [24]. Therefore, we observed that SYM affects ACC and AI´s function as target regions within the fronto-limbic circuit, which could be extended to the amygdala if we consider the detection of emotional salience and regulation, as proposed in the literature [44–47], as well as the reported connections between amygdala and medial prefrontal regions [48–52].

In line with our findings of structural and functional changes associated with SYM in ACC and insula [22–24, 26, 53], and the existing literature about amygdala [44–52], this study aims to investigate the differences in WM connections among these regions between long-term meditators of SYM and healthy controls. For this purpose, we are using diffusion weighted imaging (DWI) an MRI modality to measure microstructural WM connectivity. This technique is based on quantifying the movement of water molecules in the brain since they are restricted by the underlying arrangement of myelinated structures [54]. Several measures are derived from DWI thanks to the application of mathematical models, such as the probabilistic tractography [55], which provides an estimate of the most likely location of pathways among gray matter regions of interest. To this end, we have selected Seeds of Interest (SOIs)–ACC, AI and amygdala in both hemispheres–to analyze WM patterns among them and to extract the differences between meditators and non-meditators.

## Materials and methods

### Participants

Forty white Caucasian, healthy volunteers, participated in this research. Twenty experts in SYM (10 females), aged 47 ± 11 years (*mean* ± *SD*), were compared with 20 non-meditators (11 females), aged 46 ± 12 years.

Meditators were found among the local practitioners of SYM and assistants to a SYM seminar celebrated in Tenerife in January 2018. Meditators had between 5 and 33 years of experience of daily meditation practice in SYM (21.8 ± 7.3 years). They dedicated 76 ± 39 minutes to meditation daily. See Table 1 for demographic details.

**Table 1:** Demographic characteristics of the groups.

	Meditators *Mean (SD)*	Controls *Mean (SD)*	*t*(*df* = 38)	*p-*value*
Volunteers N°	20	20		
Age (years)	47.2 (11.1)	45.9 (11.9)	0.36	0.72
Age range (years)	19–63	22–63		
Meditation practice (years)	21.8 ± 7.3	0		
Meditation practice range (years)	5–33	0		
Meditation daily practice (minutes)	76 ± 39	0		
Education degree, 0 to 6	2.3 (1.8)	2.5 (1.7)	-0.46	0.65
Height (cm)	169.8 (9.6)	169.0 (7.6)	0.30	0.76
Weight (Kg)	69.7 (10.2)	74.0 (15.2)	-1.04	0.30
Body mass index	24.2 (3.5)	25.7 (3.9)	-1.29	0.21

* *p*-values represent group differences between meditators and controls using two-tailed independent samples t-tests.

The recruitment period started with the MRI acquisition in January 15, 2018, and finished in December 20, 2018. Prior to participation in this research, all volunteers filled in different questionnaires to validate their individual health status. All participants informed that they had no physical or mental illness, no history of neurological disorders, and no addiction to drugs, alcohol or nicotine. To ensure accuracy of differences in WM tracts between meditators and non-meditators, subjects in the control group were excluded if they engaged in any contemplative or meditation practices.

All participants signed written informed consent form before participating in the experiments. This study protocol was approved by the Human Research Ethics Committee of the University of La Laguna, Tenerife, Spain (approval number: CEIBA 2011–0023), to protect the participants’ rights according to the Declaration of Helsinki and the rules of research at the University of La Laguna.

### Imaging protocol

The sample data was recorded in a 3.0T General Electric Medical system located at the Hospital’s Magnetic Resonance Service for Biomedical Research at the University of La Laguna. The scanning protocol included a high-resolution T1-weighted MPRAGE anatomical and a series of DWI. The anatomical volumes were consisting of a total of 196 contiguous 1mm sagittal slices which were acquired with the following parameters: Repetition Time (TR) = 8.844 ms, Echo Time (TE) = 1.752 ms, Field of View (FOV) = 256 × 256 mm^2^, in-plane resolution = 1 mm × 1 mm, flip angle = 10°. The DWI images were acquired along 32 independent directions. The scan protocol set 64 slices spaced at 2.4 mm, with 2 mm x 2 mm in-plane resolution and a diffusion weighting b-value of 1000 s/mm^2^. One reference image (b_0_ image) with no diffusion weighting was also obtained (b = 0 s/mm^2^). The following parameters were used: FOV = 128 x 128, TE = 71.7 ms, TR = 17000 ms, flip angle = 90°.

### Preprocessing and Seeds of Interest

**Fig 1:**
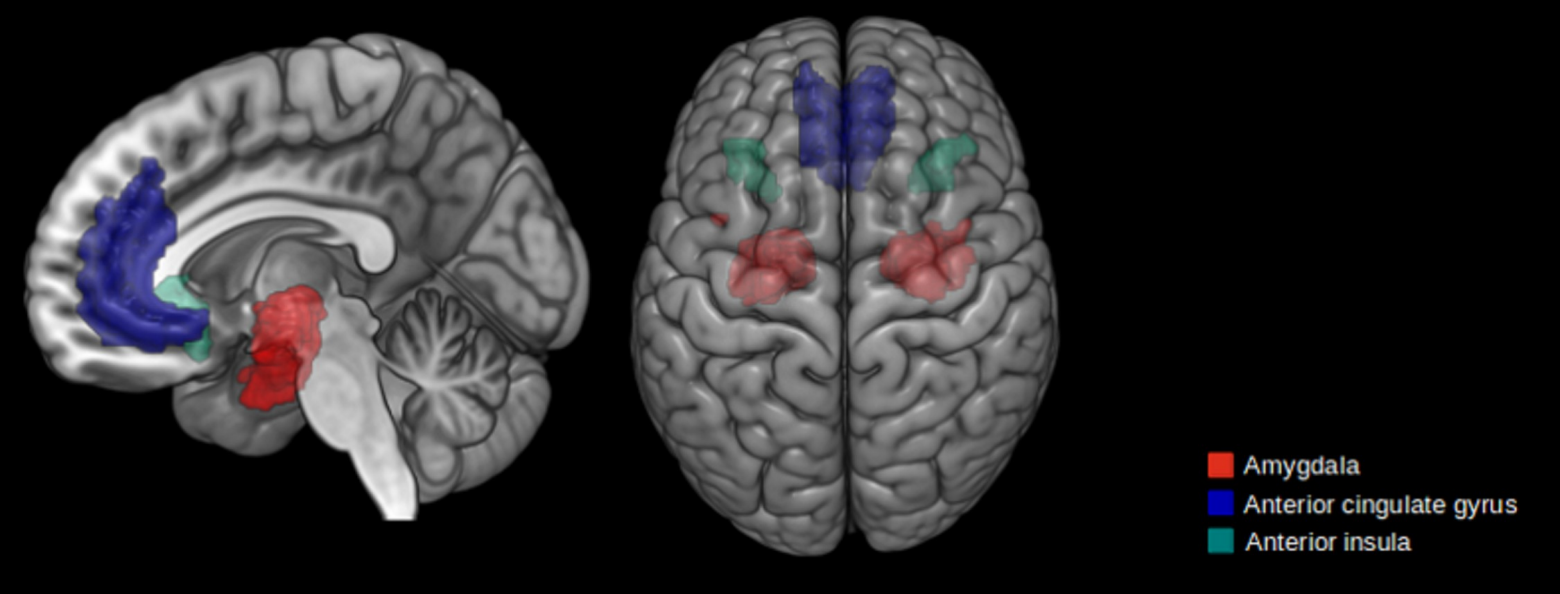
Seeds of interest used for probabilistic tractography. The seeds were projected on the inflated surface of a control subject.

The anatomical images were aligned automatically by the ACPCDETECT program [56] which is a module of the Automatic Registration Toolbox. The program takes a 3D T1-weighted structural MRI of the human brain as input and automatically detects the mid-sagittal plane, then detects the Anterior Commissure and Posterior Commissure intersection points and finally, detects 8 additional landmarks (the so-called Orion landmarks) on the mid-sagittal plane. This information was used to tilt-correct the input volume into a standard orientation. Each realigned-T1 was submitted to Freesurfer v6.0 [57], where the function recon-all performed tissue classification and anatomical labeling based on Destrieux’s Atlas [58, 59]. The anatomical labeling allowed the selection in both hemispheres of the seeds of interest (SOIs): ACC, AI, and amygdala (Fig 1).

### Diffusion tensor imaging processing

All processing steps for diffusion tensor imaging (DTI) were performed using the Functional MRI of the Brain Software Library version 6 (http://www.fmrib.ox.ac.uk/fsl/) [60]. First, the eddy current-induced distortion correction was performed, and later, we executed the probabilistic tractography [55] using FMRIB’s Diffusion Toolbox to produce an estimate of the most likely location of pathways among the SOIs of gray matter. Therefore, a local model of fiber orientation, capable of resolving crossing fibers, was inferred from the data, followed by building up distributions on the diffusion parameters at each voxel in the individual subject’s space by repetitive sampling, 5000 sample tracts were generated from each seed voxel.

The selected option to perform the tracking was voxel by SOI connectivity. This methodology estimates the voxel-SOI connectivity by quantifying the connectivity values between each voxel in a seed mask and any number of user-specified target masks. Then each SOI was used as seed (setting the rest SOIs as targets) and the resulting connectivity matrix for each seed was normalized dividing by the maximum value of estimated fibers for that seed.

### Statistical analysis

The analysis was conducted with SPSS (IBMCorp. V.27). We ran a MANOVA analysis and examined the estimated marginal means of the model, which were corrected for multiple comparisons (Bonferroni), to test the effects of meditation group on probabilistic connectivity within pairs of SOIs. These pairs comprised six SOIs, three in the left hemisphere (Left ACC, Left AI, Left amygdala) and three in the right hemisphere (Right ACC, Right AI, Right amygdala).

As previously mentioned, all six SOIs were used as seeds, where the probability tracking started to construct possible paths, while the remaining five SOIs were considered as targets where the tracking could finish. This resulted in a total of 30 possible paths: six SOIs multiplied by five possible target SOIs for each seed.

## Results

The MANOVA analysis showed a significant multivariate model for group effect (*F*(30,9) = 3.795, *p* = 0.020).

**Fig 2:**
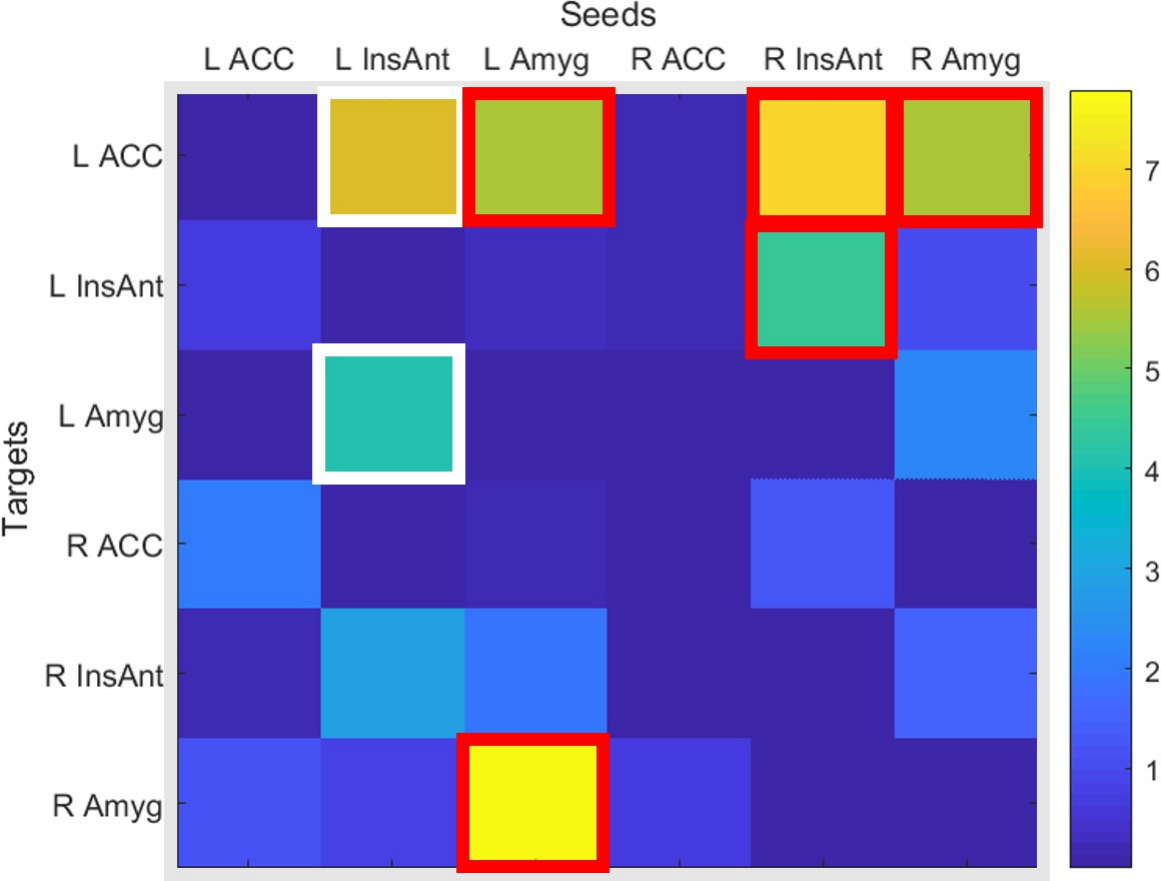
Pseudo color *F-*value among all seeds and targets, significant (*p*<0.05, Bonferroni corrected) are those 2 paths framed in white squares where controls have more tracts and framed in red squares the 5 paths where meditators have more tracts.

The results of the differentiations of pathways between groups showed that there were 7 paths in which there were significant differences between meditators and controls, in 5 out of 7 paths, meditators showed larger number of tracts while controls showed larger number of tracts in 2 of the paths (see Table 2 and Fig 2).

**Table 2:** Statistically significant results among seeds and targets.

Seed–Target	*Contr. Mean	*Medit. Mean	**Medit.-Contr.	95% confidence interval for the difference^a^		*SE*	Sig.^a^
				Lower Bound	Upper Bound		
Left amygdala–Right amygdala	0.101	0.155	0.054	0.015	0.093	0.019	0.008
Right AI–Left ACC	0.000	0.002	0.002	0.000	0.003	0.001	0.012
Left amygdala–Left ACC	0.699	0.755	0.056	0.008	0.103	0.024	0.023
Right amygdala–Left ACC	0.009	0.027	0.018	0.003	0.034	0.008	0.024
Right AI–Left AI	0.000	0.003	0.003	0.000	0.006	0.002	0.042
Left AI–Left ACC	0.561	0.469	-0.092	-0.167	-0.016	0.037	0.018
Left AI–Left amygdala	0.001	0.000	-0.001	-0.002	-4.634e-6	0.001	0.049

*Contr. Mean and Med. Mean are the normalized average of tracts of each group

**Med-Con is the subtraction of Med. Mean minus Contr. Mean to see which group has more tracts, positive when meditators have more tracts.

^a^Adjusted for multiple comparisons: Bonferroni

The results show a larger connectivity in mediators relative to non-meditators in five paths, three of them having left ACC as a target (right AI–left ACC, left amygdala–left ACC and right amygdala–left ACC), one between left and right AI; and one between left and right amygdala. On the other hand, controls had larger connectivity in two paths with seed in the left AI in both cases with targets also in the left hemisphere (left AI–left ACC and left AI–left amygdala).

Four out of five paths that were larger in meditators were connecting regions across both hemispheres: two of them connecting the same area in both sides (left amygdala–right amygdala and right AI–left AI), and the other two having left ACC as Target (right AI–left ACC and right amygdala–Left ACC); and the fifth path connected two regions of the same hemisphere (left amygdala–left ACC).

It is interesting to note that there were no significant differences in paths connecting the right hemisphere areas, see bottom-right 3x3 squares at Fig 2. Also interesting was that the 2 paths in which controls had larger connectivity were between left hemisphere areas departing both from left AI, see up-left 3x3 squares at Fig 2.

### Correlation analyses

No significant correlations were found within the meditation group between pathways that significantly differed between groups and the meditators’ experience. Additional analyses were conducted for the average time per day dedicated to meditation and the frequency of perception of MS. The only within group correlation observed was between the left AI–left ACC pathways and the frequency of perception of MS in meditators (*r* (18) = -0.444, *p* = 0.05). However, this effect does not survive corrections for multiple comparisons and is not further discussed.

## Discussion

Our study shows that extended regular practice of SYM is associated with WM fiber connections in the fronto-limbic circuits. In expert SYM practitioners, we found enhanced connectivity between the right AI and bilateral amygdalae with the left ACC (right AI–left ACC, left amygdala–left ACC, and right amygdala–left ACC). Additionally, increased connectivity was observed in the interhemispheric connectivity between right and left AI and right and left amygdalae. In contrast, controls showed increased connectivity in two paths originating from the left AI, one connecting the left AI with the left amygdala (left AI–left amygdala) and the other connecting the left AI with the left ACC (left AI–left ACC).

Therefore, it appears that the right and left AI showed opposite patterns of connectivity with the ACC in SYM experts. Furthermore, increased connectivity within the fronto-limbic circuit shows that SYM may involve the ACC as well as inter-hemispheric patterns, while showing reduced connectivity from left AI. Interestingly, this pattern of opposite connectivity robustness relative to controls in different fronto-limbic structures may be related to top-down emotion regulation in expert SYM [44–46].

Meditators showed larger structural connectivity pathways connecting the left and right amygdalae with the left ACC (left amygdala–left ACC and right amygdala–left ACC). Based on previous evidence from functional [31, 61] and structural [29, 42, 62] meditation research, we hypothesize that the observed effect is related to the top-down inhibitory regulation from ACC towards amygdala [63–65]. The modulation of the ACC over amygdala reactivity is a key factor in emotional self-regulation [66]. Furthermore, there is evidence that the harmonious interaction between amygdala and ACC appears important for cultivating good feelings, while the negative correlation between these regions plays a significant role in extinguishing negative feelings [67].

This could be related to the state of MS, thought to enhance the meditator‘s capacity to observe their feelings from a meta-cognitive, detached witness state, which could be a key to regulate and control their emotions [68, 69]. Additionally, in rumination related to one’s feelings, the amygdala is more active during thinking and more deactivated during the feeling condition [70]. Therefore, through emotion-introspection, a process which has been shown to deactivate the amygdala [70], and presumably through an automatic emotion regulation [71, 72], this state of MS may promote increased WM connectivity between the ACC and the amygdala and could be a key to enhanced emotional control and regulation effects associated to meditation practice.

In addition, our findings align with studies of other meditation techniques that showed differences in the WM tracts between ACC and amygdala [29, 42], which were suggested to reflect the reappraisal of emotions through the top-down modulation of the amygdala by the ACC and prefrontal areas [61, 62]. Furthermore, increased FC between left amygdala and dorsal ACC was found during emotion processing in experts of loving-kindness meditation (Theravada tradition), which was attributed as part of the downregulation of the fear response [67]. Similarly, mindfulness meditation appears to be associated with increased connectivity in ACC regions during emotion regulation related to self-control [73].

Several studies show that a malfunction in the ACC–amygdala connectivity could be associated with anxiety, depression, bipolar disorder and other personality disorders. For instance, in patients with anxiety disorders, threatening images promote greater reactivity in both the amygdala and the ACC, suggesting increased effort required to regulate fear arousal [74]. Brain anatomy studies have shown an important bidirectional connectivity path between the amygdala and PFC areas [75, 76], including the ACC, that is deteriorated in anxiety [66] and bipolar disorder [77]. Therefore, interrupting the flow of negative thoughts appears to be an important paradigm to achieve, aiming to ameliorate the deterioration associated with these mental issues. Moreover, increased activity of the ACC associated with meditation has been suggested as a potential tool for treatment of addictions that are associated with a deficiency of fronto-limbic self-control [73].

The connections from the left AI (left AI–left ACC and left AI–left amygdala), where controls had more tracts than meditators, may be linked to previous effects observed on reward processing and interference inhibition in meditators [25, 78]. These effects were related to the left AI role in decision-making processes and anticipation of negative outcomes. Previous findings, such as reduced interference inhibition during the Simon task and reduced FC between left insula and mid-cingulate in SYM practitioners [25], have been associated to faster conflict resolution following meditation training [79–81]. Additionally, during incentive anticipation, meditators exhibited reduced FC between right caudate and bilateral AI [78]. Hence, we could hypothesize that SYM training may influence structural WM connections in a way that improves emotional regulation and cognitive control during decision making and reward processing.

On the other hand, meditators had better connectivity between right AI and left ACC and between right AI and left AI. This expands upon our previous findings, where the activity of right AI was associated with the establishment of MS [23]. Furthermore, the right AI appeared to be functionally more connected with ACC in association with deeper MS experienced inside the MRI scanner [26]. Moreover, the right AI was found to be more activated and structurally larger in individuals after 4 weeks of meditation training [53], as well as structurally larger in long-term SYM [24]. Therefore, our results align with the attributed role of this area in inner awareness or interoception and as a pivotal region of the brain’s attention systems [82–85]. It is interesting to remark the asymmetric results obtained from both AI, where meditators had more connections on the pathways departing from the right AI (right AI–left ACC and right AI–left AI) while controls had more connections when seeding the left AI (left AI–left ACC and left AI–left amygdala). This is probably related to the functions of the right AI previously indicated (inner awareness or interoception) [82–85], which are more demanded by practitioners of SYM in their regular practice of meditation. It is also important to notice that right AI, at both functional and structural levels, has been linked with more brain areas than the left AI [85–87]. Additionally, it has been suggested that the right insula serves as a crucial node between the central executive/attentional network and the default mode network [85, 88–90], which may play an important role in meditators of SYM practicing MS. On the other hand, the left AI functions are related with language tasks, like speech production or inner speech [91, 92]. These functions depict the left AI as an area that is probably recruited to a smaller extent by a less or non-thinking brain, such as those of meditators of SYM (for a review of the structural and functional asymmetry of the AI see [85, 93]).

Insular effects have been observed across different types of meditation. DTI research in expert meditators of Buddhist and Zen traditions, revealed increased fractional anisotropy in the bilateral insula when comparing meditators to controls [42]. Other types of meditation, mostly mindfulness meditation, have shown consistent gray matter effects over the right insula [38, 41, 62, 94, 95], which was associated with increased awareness of internal states [94, 95] and the integration of bottom-up interoceptive signals with top-down predictions [42, 96] possibly promoting awareness in the meditative state.

Some important limitations in our study are the use of SOIs, which limits the analysis of possible areas that could be involved in meditation. However, we have focused on a set of *a priori* important SOIs based on our previous studies, but new studies are needed to continue depicting the differences in WM tracts associated with cognitive and emotion control and regulation in SYM. On the other hand, this restriction enhanced the statistical power of our results. Additionally, the observed results may not be attributed to meditation practice exclusively, since other uncontrolled differences between groups, such as variations in diet, physical activity, or exposure to harmful pollutants, could also occur. The ideal study design would be a longitudinal randomized controlled trial which is, however, not feasible in cross-sectional studies that investigate the long-term effects of meditation practice on brain structure such as this one. Furthermore, the interpretation of our results is limited by the nature of DTI, which does not provide evidence for directionality. Future research could be enhanced by complementing similar analyses with recent developments such as laminar fMRI [97] to provide directionality.

## Conclusions

This is the first brain morphometry characterization of WM tracts associated with long-term practice of SYM. We show that long-term SYM meditators compared with non-meditators have significantly larger structural connectivity in five fiber tracts linking interhemispheric connections between right and left amygdalae and AI, and between left ACC with both amygdalae and right AI, which are related in the literature with top-down attention and emotion self-regulation. Enhancement of these connections could be linked with the witness state perceived during MS in SYM. On the other hand, the two tracts with higher connectivity in controls both linking left AI with left ACC and with left amygdala could be linked with reduced emotional influence over top-down control.

## Supporting information

S1 File Sahaja Yoga Meditation diagram. Diagram with the location of the chakras, the kundalini, and their qualities according to SYM.
(PDF)

S2 File (XLSX)
