# Correction: Enhanced amygdala–anterior cingulate white matter structural connectivity in Sahaja Yoga Meditators

**DOI:** 10.1371/journal.pone.0321765

**Published:** 2025-04-01

**Authors:** Oscar Perez-Diaz, Daylín Góngora, José L. González-Mora, Katya Rubia, Alfonso Barrós-Loscertales, Sergio Elías Hernández

The following information is missing from the funding statement: JLGM has received support from the Spanish Ministry of Science and Innovation (Ministerio de Ciencia e Innovación, PÍD. 126172NB-100, MEC.PHC21).
