# Supplementary material for: Enhanced amygdala–anterior cingulate white matter structural connectivity in Sahaja Yoga Meditators
Source: PLoS One. 2024 Mar 28;19(3):e0301283. doi: 10.1371/journal.pone.0301283 (PMC10977753; doi:10.1371/journal.pone.0301283)

Additional file 1:

Diagram with the location of the chakras, the kundalini, and their qualities according to SYM is provided as supplementary data.

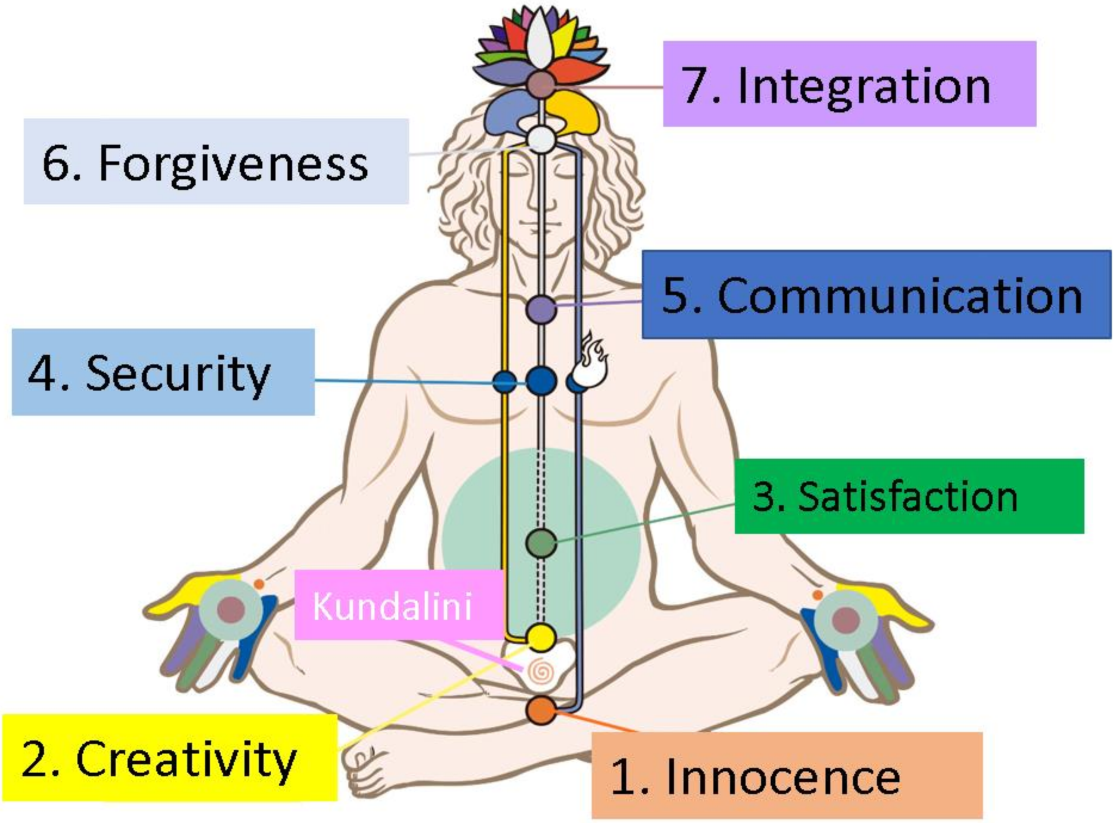

Supplement: S1 File — Diagram with the location of the chakras, the kundalini, and their qualities according to SYM. (PDF) [file pone.0301283.s001.pdf]
